# Alzheimer’s Disease-Associated Molecular Abnormalities in White Matter Glia and Related Pathologies Detected in Unfractionated and O4-Selected Serum Exosomes Using a Liquid Biopsy Approach

**DOI:** 10.3390/biomedicines14010251

**Published:** 2026-01-22

**Authors:** Suzanne M. de la Monte, Ming Tong

**Affiliations:** 1 Departments of Pathology and Laboratory Medicine, Neurology and Neurosurgery, Rhode Island Hospital, Alpert Medical School of Brown University, Brown University Health, Providence, RI 02903, USA; 2 Department of Pathology, Women & Infants Hospital, Providence, RI 02905, USA; 3 Department of Medicine, Rhode Island Hospital, Alpert Medical School of Brown University, Brown University Health, Providence, RI 02903, USA; mtong@brownhealth.org

**Keywords:** exosome, Alzheimer, white matter, oligodendrocyte, serum, extracellular vesicle, neurodegeneration, liquid biopsy, myelin, glycoprotein

## Abstract

**Background/Objectives**: White matter degeneration is a significant and early mediator of cognitive impairment in Alzheimer’s disease (AD), yet the critical pathologic features remain poorly understood, under-detected, and therapeutically untargeted. Herein, we characterize molecular features of white matter glial cells in AD brains and assess the utility of non-invasive approaches for detecting related abnormalities in extracellular vesicles (EVs) isolated from serum (SEV). In addition, results from unfractionated (SEV-T) and O4 sulfatide-selected SEVs were compared to determine whether white matter abnormalities were detected with greater sensitivity in oligodendrocyte-specific SEVs (SEV-O4). **Methods**: Oligodendrocyte glycoprotein and astrocyte mRNA levels were measured in postmortem human AD and control frontal lobe white matter by RT-PCR. Immunoreactivity to oligodendrocyte glycoproteins, astrocyte structural proteins, neurofilament light chain (NfL), and aspartyl-asparaginyl-β-hydroxylase (ASPH) was measured by ELISA in SEV-T and SEV-O4 from patients with moderate AD or normal aging. **Results**: AD brain pathology was associated with significantly reduced mRNA expression of multiple oligodendrocyte glycoproteins and increased mRNA expression of astrocytic structural genes. SEV analyses demonstrated significantly increased immunoreactivity to 2′,3′-cyclic nucleotide 3′ phosphodiesterase (CNPase), myelin-associated glycoprotein 1 (MAG1), astrocyte proteins, and ASPH, a potent activator of Notch and myelin-regulated homeostatic functions. There were no significant benefits of measuring SEV-O4 compared with SEV-T immunoreactivity. **Conclusions**: AD is associated with significant molecular abnormalities in oligodendrocyte and astrocyte function in brain tissue. The abnormalities detected in SEVs likely reflect oligodendrocyte injury and degeneration, as well as astrocytic activation. The findings suggest that low-invasive SEV approaches, including the novel analysis of ASPH upregulation, can be used to detect and monitor AD white matter degeneration.

## 1. Introduction

White matter is an important target of neurodegeneration in Alzheimer’s disease (AD), including in its early stages [1]. Cognitive impairment is mediated by inefficient neurotransmission resulting from dysfunction or loss of oligodendrocytes, myelin, axons, and synaptic integrity. Although neuroimaging can detect white matter atrophy and related abnormalities [2,3], it may not be suitable for efficient disease monitoring or prevention. On the other hand, extracellular vesicles (EVs), which are membranous nanoparticles that bear phenotypic features and molecular cargo characteristic of their cells of origin, traverse vascular networks and are detectable in biological fluids such as serum, saliva, cerebrospinal fluid, and extracellular tissue fluid [4]. A rapidly growing trend is to use EVs from body fluids for low-invasive diagnostics and disease monitoring, including for malignancies, neurodevelopmental defects, and neurodegeneration [5,6,7,8]. Bottlenecks to the successful implementation of these approaches stem from limitations in the accurate detection and validation of pathological changes. Non-neoplastic central nervous system (CNS) metabolic disorders are in dire need of novel tools to accelerate diagnoses and provide rapid, objective, quantifiable means of monitoring treatment responses. To determine whether EV-based approaches could be used to detect white matter degeneration in AD, studies are needed to compare related alterations in myelin/oligodendrocyte glycoprotein expression in brain tissue and EVs isolated from serum.

In AD, white matter oligodendrocytes and myelin exhibit striking changes in membrane glycoprotein and lipid expression, together with evidence of oxidative injury and lipid peroxidation [1]. Recent studies have shown that white matter/oligodendrocyte pathology in alcohol-related brain degeneration [9] and in an experimental model of Alzheimer’s disease [10] can be detected in EVs isolated from brain tissue and serum. Besides oligodendrocyte/myelin glycoproteins, recent evidence suggests that white matter disease is associated with altered expression of aspartyl-asparaginyl-β-hydroxylase (ASPH), an insulin/insulin-like growth factor-responsive molecule that regulates Notch pathway signaling but is also upregulated by oxidative stress via activation of hypoxia-inducible factor 1 (HIF-1α) [11]. Experimental models have demonstrated that ASPH is expressed in oligodendrocytes [12] and that ASPH expression is altered in white matter diseases with myelin degeneration [13]. Furthermore, exploratory studies revealed that ASPH immunoreactivity is detectable in SEVs (unpublished). Therefore, aberrant ASPH expression in SEVs may represent a novel marker of white matter degeneration.

Neurofilament light chain (NfL) immunoreactivity has been shown to be elevated in neurodegenerative conditions [14,15,16,17], including AD [18,19,20,21]. NfL accumulation marks neuronal injury, including axonal damage [15]. Neurodegeneration-linked elevations in NfL immunoreactivity are detectable in serum, brain tissue, and cerebrospinal fluid [14,15,16,17], and in some diseases, EVs as well [22]. Therefore, it was of interest to determine whether altered NfL immunoreactivity could serve as an additional marker of white matter degeneration using SEV-based assays.

In our attempt to characterize the utility of serum EV-based assays of white matter degeneration, we considered the potential need to refine the approach by selecting CNS-specific EV subsets for analysis. Since myelin/oligodendrocyte glycoproteins are uniquely expressed in the nervous system [23,24], it may be feasible to evaluate AD effects on myelin/oligodendrocyte glycoproteins in unfractionated SEVs. On the other hand, if greater sensitivity and specificity are required to detect AD-related changes in immunoreactivity to low-abundance myelin/oligodendrocyte molecules, analysis of oligodendrocyte-specific EVs isolated from serum may be warranted [23]. In this regard, EVs shed by degenerating oligodendrocytes bear alterations in myelin/oligodendrocyte markers reminiscent of abnormalities in white matter [7,25,26,27]. O4 sulfatide is a surface membrane marker specific for cells of the oligodendrocyte lineage [23]. Enriching EVs for the O4+ fraction may increase the sensitivity of detecting shifts in AD-related myelin/oligodendrocyte glycoprotein expression. The analysis of brain-specific markers of myelin/oligodendrocytes offers exciting opportunities to evaluate the emergence and progression of white matter pathology in AD using non-invasive assays. To this end, we designed a study to evaluate myelin/oligodendrocyte glycoprotein expression in unfractionated and O4+ SEVs, and to compare these findings with AD effects in brain tissue.

## 2. Materials and Methods

*Sample Sources:* De-identified human postmortem fresh frozen AD (n = 8) and control (n = 8) frontal lobe tissue was obtained from the Duke Kathleen Price Bryan Brain Bank and Biorepository at Duke University (Durham, NC, USA) Medical Center (RRID:SCR_005022). Information on age, sex, and apolipoprotein E (APOE) genotype was provided by the brain bank. A standardized brain banking protocol ensured storage of high-quality tissue for molecular and biochemical analyses, apolipoprotein E genotype determination, and AD Braak Score assessment. The cases included in this study had an APOE ε3/ε3 genotype and a Braak stage score of 0–2 (control) or 5–6 (advanced AD). An AD diagnosis was rendered using standard neuropathological criteria for the postmortem cases [28]. De-identified and coded AD and control remnant clinical serum samples (9 AD and 9 controls) from patients unrelated to the postmortem cases were stored at −80 °C in the Brown University Health Molecular Neuropathology Research laboratory. The NINCDS-ADRDA criteria were used to establish the clinical diagnosis of AD [29]. All specimens (postmortem brain and serum) were obtained with written informed consent for their use in future research. The use of deidentified human postmortem tissue for research meets Exemption Criteria 4 under 45CFR Part 46 (Project 2029341-1, approved 26 June 2023). The Brown University Health (formerly Lifespan) Institutional Review Board (IRB) approved the use of de-identified human samples for research (Protocol code #413318 45 CFR 46.110; date of approval on 30 August 2018).

*Targeted Quantitative Reverse Transcriptase Polymerase Chain Reaction Array:* Total RNA was isolated from 100 mg samples of fresh-frozen AD and control frontal lobe tissue using the RNeasy Mini Kit (Qiagen, Hilden, Germany), and reverse transcribed using the AMV 1st Strand cDNA Synthesis Kit (Roche, Indianapolis, IL, USA). The resulting cDNA templates were used to measure mRNA transcripts encoding 10 genes, namely immature or non-myelinating oligodendrocyte glycoproteins ((2′,3′-cyclic nucleotide 3′ phosphodiesterase (*CNP*), group-specific component vitamin D binding protein (*GALC*), proteolipid protein (*PLP*), and platelet-derived growth factor-alpha (*PDGFR-α*)); mature oligodendrocyte/myelin glycoproteins (myelin oligodendrocyte glycoprotein (MOG), myelin-associated glycoprotein 1 (MAG1), myelin basic protein (MBP)); astrocyte structural proteins ((Nestin, Vimentin, and glial fibrillary acidic protein (GFAP)); and hypoxanthine phosphoribosyltransferase 1 (*HPRT1*) as the internal control (see Supplementary Table S1 for gene functions). The PCR primer pairs were designed using Primer3 software (Release 2.6.0) (http://primer3.sourceforge.net/, accessed on 27 January 2022) (Supplementary Table S2). Targeted arrays were constructed by spotting and drying primer pairs (10 pmol/10 µL) into individual wells of a Lightcycler 480 Multi-well Plate 96 (Roche, Indianapolis, IN, USA). The arrays were sealed and stored at −80 °C. On the day of use, the array plates were thawed on ice, and 20 µL of reaction cocktail containing cDNA from a 2 µg RNA template and Sybr green master mix was added to each well. The qPCR reactions were performed using a Roche Lightcycler 480 System [30]. Relative mRNA abundance was calculated using the 2^−ΔΔCt^ method, and the results were normalized to internal controls (hypoxanthine–guanine phosphoribosyltransferase (*HPRT*), ribonuclear protein, RNP, and beta-actin (*Actβ*)).

*EV Isolation*: Total EVs were isolated from serum using the Miltenyi Biotec Exosome Isolation Kit (Miltenyi Biotec). The samples (650 µL) were first diluted 1:1 with phosphate-buffered saline (PBS) and then centrifuged at 2000× *g* for 30 min to remove cellular debris. The resulting supernatants were transferred to fresh tubes and centrifuged at 10,000× *g* for 45 min. Magnetically labeled Exosome MicroBeads Pan (CD9, CD63, and CD81) (50 µL/sample) were added to the clarified samples, vortex-mixed, and incubated at room temperature for 1 h. After preparing the columns and placing them in the magnetic field of the MicroMACS Separator attached to a MACS MultiStand according to the manufacturer’s protocol, the magnetically labeled samples were applied to the columns under a strong magnetic field. After washing the columns, the magnetic field was released, and the bound EVs were recovered by elution. To isolate O4+ EVs, the clarified serum samples were incubated with magnetic Anti-O4 MicroBeads and processed as detailed above. Nanoparticle tracking analysis with ZetaView, Version 8.05.16 SP7 Particle Metrix GmbH, Ammersee, Germany) was used to confirm nanoparticle size profiles (20–250 nm) and concentrations against polystyrene nanoparticle standards.

*Serum-EV Characterization:* The EV samples were characterized using direct binding enzyme-linked immunosorbent assays (ELISAs) to measure immunoreactivity to immature and non-myelinating oligodendrocyte glycoproteins (CNP, GALC, PLP1, and PDGFRA), mature oligodendrocyte/myelin glycoproteins (MOG, MAG, MBP), astrocyte structural proteins (Nestin, Vimentin, and GFAP), neurofilament light chain (NfL), aspartyl-asparaginyl-β-hydroxylase (ASPH), tetraspanins (CD9 + CD63 + CD81), large acidic ribosomal protein (RPLPO), and heat shock protein 70 (HSP70). See Table S3 for antibody information. The assays were performed in triplicate. To perform the ELISAs, the EV samples were solubilized in lysis buffer containing 50 mM Tris (pH 7.5), 150 mM NaCl, 5 mM EDTA (pH 8.0), 50 mM NaF, 0.5% Triton X-100, and protease inhibitor cocktail (1 mM PMSF, 0.1 mM TPCK, 2 µg/mL aprotinin, 2 µg/mL pepstatin A, 1 µg/mL leupeptin). To ensure complete EV lysis, the samples were thoroughly mixed with buffer and incubated on ice for 15 min. Protein concentrations were measured with the gicinchoninic acid (BCA) assay. For the direct binding ELISAs, 50 ng of EV sample protein were diluted in 50 µL bicarbonate binding buffer and adsorbed to the bottom surfaces of 96-well MaxiSorp plates overnight at 4 °C. Non-specific binding sites were masked with Superblock TBS. The samples were incubated with primary antibodies (0.2–5.0 µg/mL) overnight at 4 °C. Immunoreactivity was detected with horseradish peroxidase-conjugated secondary antibodies, and Amplex UltraRed soluble fluorophore was measured in a SpectraMax M5 microplate reader (Ex 530 nm/Em 590 nm). The calculated ratios of target protein to tetraspanin cocktail (CD9 + CD63 + CD81) were used for inter-group statistical comparisons.

*Data Analysis:* The results were analyzed and graphed using GraphPad Prism 10.5 (San Diego, CA, USA). Violin plots depict the medians (mid-horizontal bars), first (lower horizontal lines), and third (upper horizontal lines) quartiles, and ranges (extremities) in immunoreactivity. Inter-group comparisons were made by one-way or two-way analysis of variance (ANOVA) and post hoc Tukey tests. Heatmaps compared inter- and intra-group differences in analyte expression, which were analyzed using two-way ANOVA and post hoc Tukey tests. Statistical significance was set at *p* ≤ 0.05. In addition, because among the clinical cases, the controls were significantly younger than the AD group, a multivariate analysis of variance (MANOVA) using the Number Cruncher Statistical Software (NCSS 2021; Kaysville, UT, USA) was conducted to assess how age affected white matter mRNA and immunoreactivity.

## 3. Results

Donor Characteristics: Postmortem frontal lobe samples were obtained from 8 controls and 8 AD cases. The postmortem diagnoses were established using standard criteria [28]. Controls had Braak Stage AD scores of 0–2, and the AD group had Braak Stage scores of 5 or 6, i.e., advanced (Table 1). There were no significant inter-group differences in mean age. Each group included 4 males and 4 females.

Human serum samples used for the EV studies were from 9 control and 9 AD donors (Table 2). The proportions of males and females were similar; however, the AD group was significantly older (*p* < 0.0001). The mean Mini-Mental State Exam (MMSE) score reflected moderately severe dementia in the AD group. Controls were not tested (NT). Serum Aβ_1–42_ and pTau (pT307) levels measured at the Brown University Health laboratory with commercial ELISAs were significantly lower in the AD group. However, parallel cerebrospinal fluid (CSF) revealed higher pTau (pT307) and CSF/Serum pTau (pT307) ratios in the AD group [31], supporting the clinical diagnosis of AD [29]. The original CSF samples were exhausted and, therefore, were no longer available for use in the present study.

**Figure 1:**
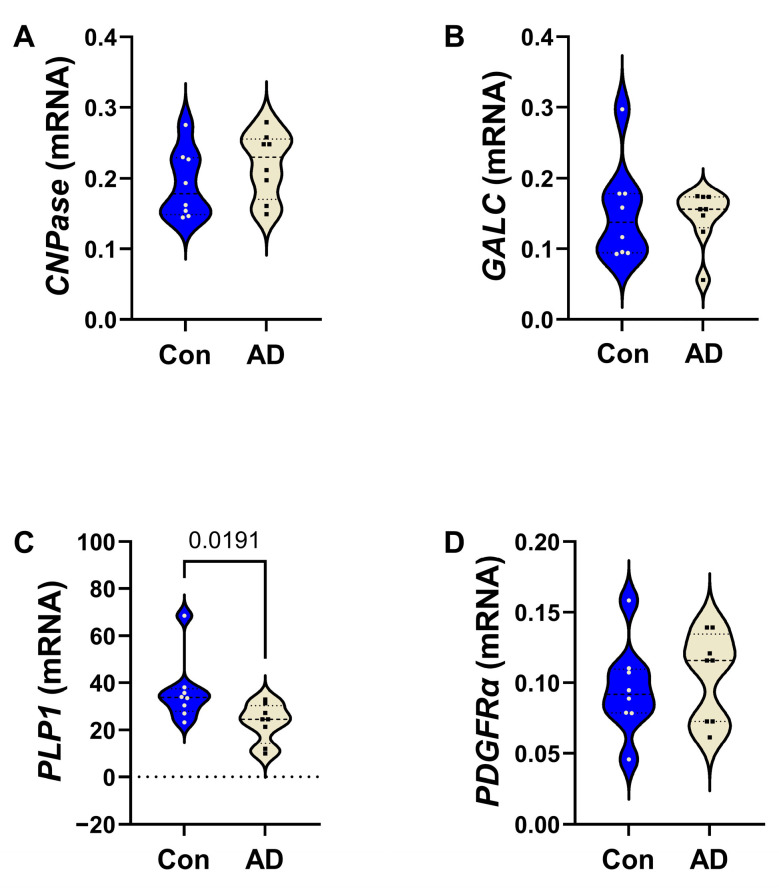
AD effects on immature and non-myelinating oligodendrocyte glycoprotein mRNA expression in frontal lobe white matter. Control (n = 8) and AD (n = 8) mRNA transcripts were measured by qRT-PCR using a Roche LightCycler 480 System. Violin plots (mean, quartiles, and range) display results corresponding to (**A**) *CNPase*, (**B**) *GALC*, (**C**) *PLP1*, and (**D**) *PDGFR-α.* Relative mRNA abundance was calculated using the 2^−ΔΔCt^ method with results normalized to internal control genes. Data were analyzed by two-way ANOVA with post hoc Tukey multiple-comparison tests. Significant (*p* ≤ 0.05) differences are shown within the panels. See Supplementary Tables S1 and S2 for the full gene names, PCR primer sequences, and gene functions.

**Figure 2:**
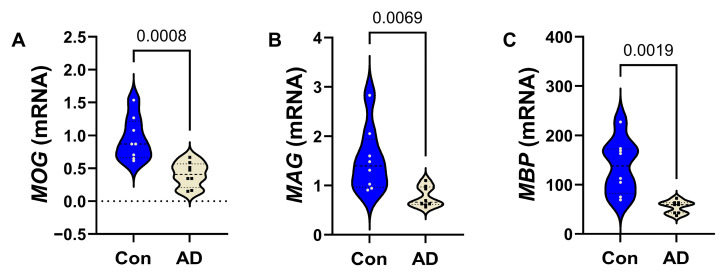
AD effects on mature oligodendrocyte/myelin glycoprotein mRNA expression in frontal lobe white matter. Control (n = 8) and AD (n = 8) purified RNA was reverse-transcribed to generate cDNA, and gene expression was measured by qRT-PCR using a Roche LightCycler 480 System. Violin plots (mean, quartiles, and range) display results corresponding to (**A**) *MOG*, (**B**) *MAG*, and (**C**) *MBP.* Relative mRNA abundance was calculated using the 2^−ΔΔCt^ method with results normalized to internal control genes. Data were first analyzed by two-way ANOVA followed by post hoc Tukey multiple-comparison tests. The corresponding significant (*p* ≤ 0.05) differences are shown within the panels. See Supplementary Tables S1 and S2 for the full gene names, PCR primer sequences, and gene functions.

**Figure 3:**
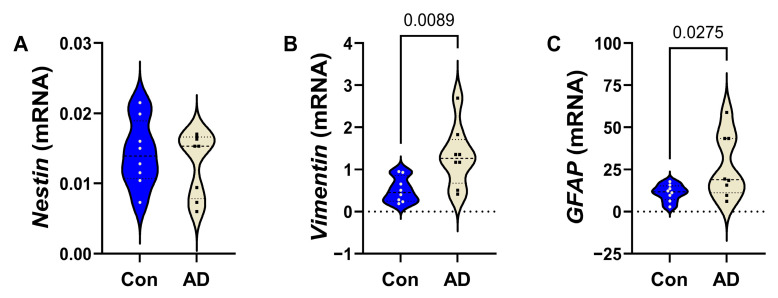
AD effects on expression of astrocytic mRNA transcripts in frontal lobe white matter. Control (n = 8) and AD (n = 8) purified RNA was reverse-transcribed to generate cDNA, and gene expression was measured by qRT-PCR using a Roche LightCycler 480 System. Violin plots (mean, quartiles, and range) display results corresponding to (**A**) *Nestin*, (**B**) *Vimentin*, and (**C**) *GFAP.* Relative mRNA abundance was calculated using the 2^−ΔΔCt^ method with results normalized to internal control genes. Data were first analyzed by two-way ANOVA followed by post hoc Tukey multiple-comparison tests. The corresponding significant (*p* ≤ 0.05) differences are shown within the panels. See Supplementary Tables S1 and S2 for the full gene names, PCR primer sequences, and gene functions.

*AD Effects on Frontal Lobe White Matter Glial mRNA Expression*: All mRNA measurements were initially compared using a two-way ANOVA, which detected significant effects of biomarker (mRNA) (*p* < 0.0001) and biomarker x diagnosis interactions (*p* = 0.0023), and a statistical trendwise effect for AD diagnosis (*p* = 0.084). The results of the post hoc multiple-comparison tests used to compare control versus AD mRNA levels of immature and non-myelinating glycoproteins (Figure 1), mature oligodendrocyte/myelin glycoproteins (Figure 2), and astrocytic markers (Figure 3) are depicted with violin plots. Analysis of the immature oligodendrocyte/myelin glycoprotein mRNA transcripts revealed that AD significantly reduced *PLP* (*p* = 0.0207) (Figure 1C), whereas *CNP* (Figure 1A), *GALC* (Figure 1B), and *PDGFR-α* (Figure 1D) were similar in the AD and control samples. AD was associated with significantly lower levels of the three mature oligodendrocyte/myelin glycoprotein mRNAs examined, including *MOG* (Figure 2A), *MAG* (Figure 2B), and *MBP* (Figure 2C).

Finally, among the astrocytic markers, AD was associated with significantly increased *VIMENTIN* (Figure 3B) and *GFAP* (Figure 3C), but no alteration in *NESTIN* (Figure 3A).

**Figure 4:**
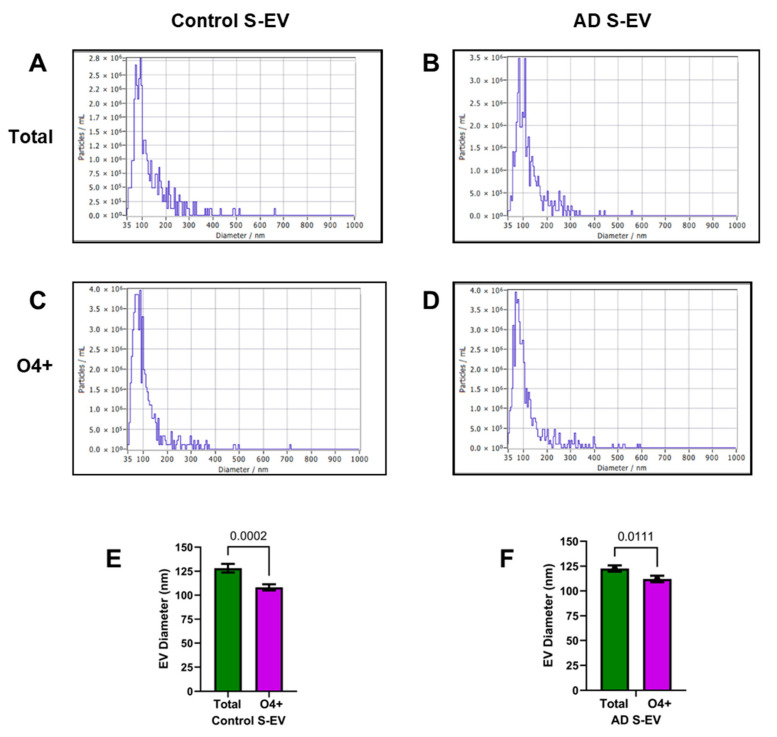
ZetaView nanotracker analysis of size (nm) x concentration (particles/mL) profiles of (**A**,**B**) unfractionated (total) and (**C**,**D**) O4+ EVs isolated from (**A**,**C**) control and (**B**,**D**) AD human serum. Among the unfractionated or O4+ EV populations, there were no significant differences in S-EV size or concentration. However, the mean diameters of both (**E**) control and (**F**) AD O4+ EVs were significantly smaller than the corresponding unfractionated EVs, as depicted in the graphs. Inter-group comparisons were made by the Welch *t*-test.

*EV Profiles*: ZetaView nanoparticle analysis determined EV size distributions, concentrations, and qualitative features, reflecting suitability for further study. The studies were used to determine if disease state or EV source impacted the size distribution profiles or mean diameters of EVs isolated from serum and brain tissue. Qualitative assessments revealed samples that were generally clean, free of debris and large aggregates, and contained few or no large EVs that would correspond to the size of apoptotic bodies. Two-way ANOVA revealed significant effects of EV type/source (*p* < 0.0001) and not diagnosis or interactive effects. The quantitative analyses demonstrated that the total/unfractionated serum EV populations (SEV-T) were significantly larger than the O4+ EVs (SEV-O4) in both control and AD cases. The size differences were due to narrower SEV-O4 peaks below 150 nm (Figure 4). There were no significant effects of AD on the SEV-T or SEV-O4 profiles or mean diameters (Figure 4).

**Figure 5:**
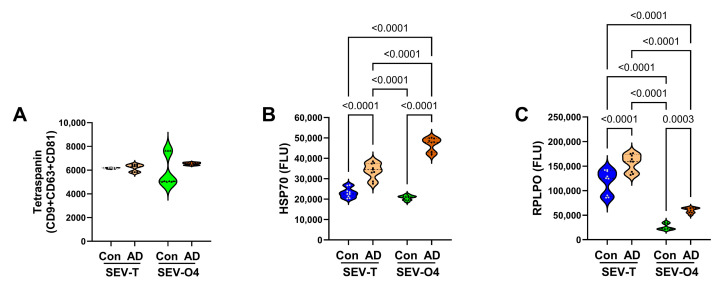
Comparative studies of (**A**) tetraspanins, (**B**) HSP70, and (**C**) RPLPO immunoreactivity measured in control (Con; n = 9) and AD (n = 9) unfractionated (SEV-T) and O4+ (SEV-O4) exosomes isolated from human serum. Immunoreactivity was measured by ELISA. Inter-group comparisons were made by ANOVA (see Table 3) and post hoc Tukey tests. Software-calculated significant *p*-values (*p* < 0.05) are displayed. HSP70 = heat shock protein 70; RPLPO = acidic ribonuclear protein. Since only the pooled tetraspanin immunoreactivity was not significantly altered by EV fractionation or AD, it was used as a normalizing control for glial and other specific protein ELISAs.

*Control Molecule EV Expression:* To determine which internal control would be most suitable for normalizing the immunoreactivity results, we compared the tetraspanin cocktail (CD9 + CD63 + CD81), HSP70, and RPLPO in control and AD SEV-T and SEV-O4 samples. A tetraspanin cocktail was used because exploratory studies showed that the expression of individual tetraspanins varied with disease state, whereas the composite assays correlated with sample protein concentration. The ELISA results demonstrated similar mean levels of tetraspanins (CD9 + CD63 + CD81) in control and AD SEV-T and SEV-O4 samples (Figure 5A; Table 3). In contrast, the ANOVA tests demonstrated significantly higher levels of HSP70 (Figure 5B) and RPLPO (Figure 5C) in AD SEV-T and SEV-O4 relative to corresponding controls. The highest level of HSP70 was observed in the AD SEV-O4, followed by the AD SEV-T samples. RPLPO expression was higher in SEV-T than in SEV-O4, and both preparations showed higher levels of RPLPO immunoreactivity in the AD group. The results of these studies showed that the tetraspanin cocktail was the most effective control for normalizing EV ELISA results for specific markers.

**Figure 6:**
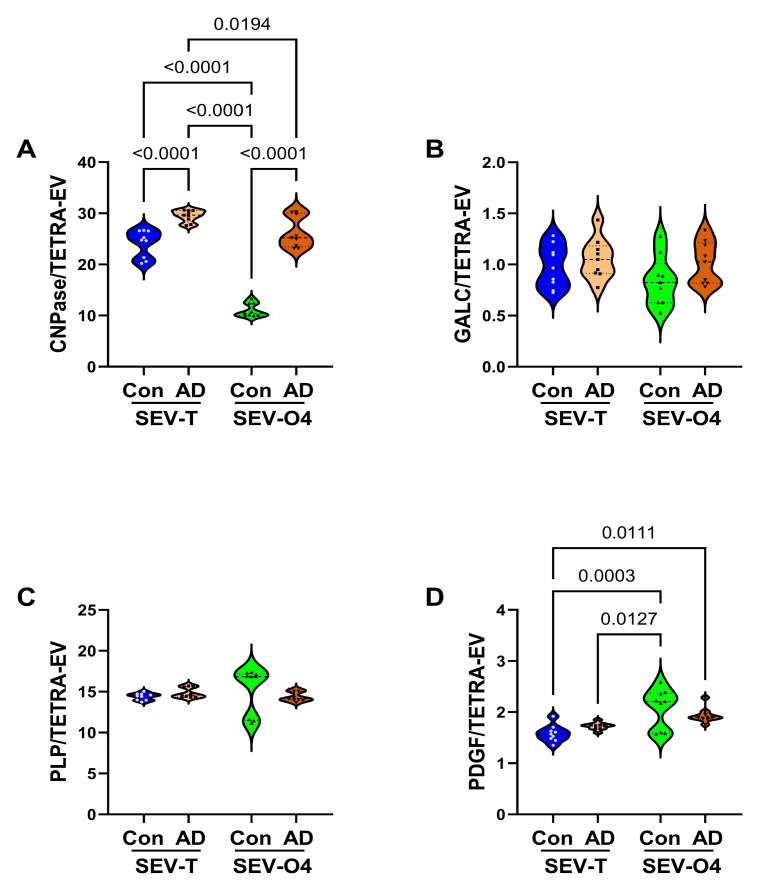
AD effects on immature and non-myelinating oligodendrocyte glycoproteins. ELISAs measured immunoreactivity to (**A**) CNPase, (**B**) GALC, (**C**) PLP, and (**D**) PDGFRα in unfractionated/total) (SEV-T) and O4+ (SEV-O4) EVs from control (n = 9) and AD (n = 9) cases. The results displayed reflect immunoreactivity normalized to tetraspanins measured with an antibody cocktail (CD9 + CD63 + CD81). Inter-group comparisons were made by ANOVA (see Table 3), and significant software-calculated *p*-values (*p* < 0.05) corresponding to post hoc Tukey tests are displayed.

*Immature Oligodendrocyte/Myelin Glycoproteins*: CNPase, GALC (Figure 6A), PLP, and PDGFR-α are clustered in this group. One-way ANOVA demonstrated significant inter-group differences for CNPase and PDGFR-α, but not GALC or PLP (Table 3). Post hoc Tukey tests demonstrated that CNPase immunoreactivity was significantly elevated in the AD SEV-T and SEV-O4 relative to corresponding controls. In addition, control SEV-O4 CNPase was significantly lower than in the other three groups. CNPase was the only immature glycoprotein in which specific AD effects were observed in both SEV-T and SEV-O4 samples, and although the inter-group difference was greater for the O4 samples, the AD effects detected with either SEV-T or SEV-O4 were essentially the same. The mean levels of GALC (Figure 6B) and PLP (Figure 6C) were similar across the groups and sample sources. Correspondingly, the ANOVA tests for GALC and PLP were not statistically significant. The significant differences in PDGFR-α (Table 1) were due to higher immunoreactivity in the control and AD SEV-O4 relative to the SEV-T samples, rather than specific effects of AD (Figure 6D).

**Figure 7:**
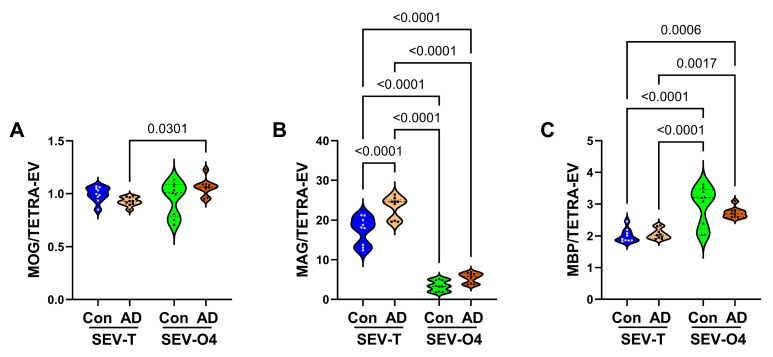
AD effects on mature oligodendrocyte glycoproteins. ELISAs measured immunoreactivity to (**A**) MOG, (**B**) MAG, and (**C**) MBP in unfractionated/total) (SEV-T) and O4+ (SEV-O4) EVs from control (n = 9) and AD (n = 9) cases. The results displayed reflect immunoreactivity normalized to tetraspanins measured with an antibody cocktail (CD9 + CD63 + CD81). Inter-group comparisons were made by ANOVA (see Table 3), and significant software-calculated *p*-values (*p* < 0.05) corresponding to post hoc Tukey tests are displayed.

*Mature Oligodendrocyte/Myelin Glycoproteins*: ANOVA tests of the mature oligodendrocyte/myelin glycoproteins revealed significant inter-group differences for MOG, MAG1, and MBP (Table 1). The violin plots and post hoc Tukey tests demonstrated significantly higher levels of MOG in AD SEV-O4 relative to AD SEV-T (Figure 7A), and significantly higher levels of MBP in control and AD SEV-O4 relative to control and AD SEV-T (Figure 7C), but no specific AD effects. In contrast, in the SEV-T sample, MAG1 immunoreactivity was significantly higher in AD relative to control (*p* < 0.0001) (Figure 7B), and both were approximately tenfold higher than in the SEV-O4 samples. In contrast to the SEV-T results, there was no significant AD effect on MAG1 in the SEV-O4 samples.

**Figure 8:**
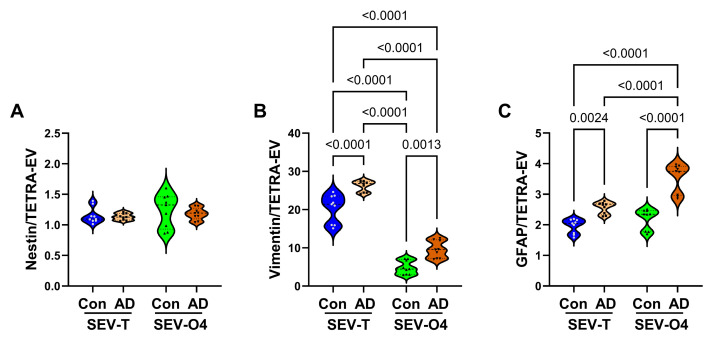
AD effects on astrocytic proteins. ELISAs measured immunoreactivity to (**A**) Nestin, (**B**) Vimentin, and (**C**) GFAP in unfractionated/total) (SEV-T) and O4+ (SEV-O4) EVs from control (n = 9) and AD (n = 9) cases. The results displayed reflect immunoreactivity normalized to tetraspanins measured with an antibody cocktail (CD9 + CD63 + CD81). Inter-group comparisons were made by ANOVA (see Table 3), and significant software-calculated *p*-values (*p* < 0.05) corresponding to post hoc Tukey tests are displayed.

*Astrocyte Proteins*: ANOVA tests were significant for vimentin (*p* < 0.0001) and GFAP (*p* < 0.0001) but not nestin (Table 3). Correspondingly, the mean levels of nestin broadly overlapped across the four sample sets (Figure 8A). AD was associated with significantly higher levels of both SEV-T and SEV-O4 vimentin (Figure 8B) and GFAP (Figure 8C). In addition, the mean levels of vimentin were significantly lower in SEV-O4 compared with SEV-T, and the highest level of GFAP was measured in AD SEV-O4 samples.

**Figure 9:**
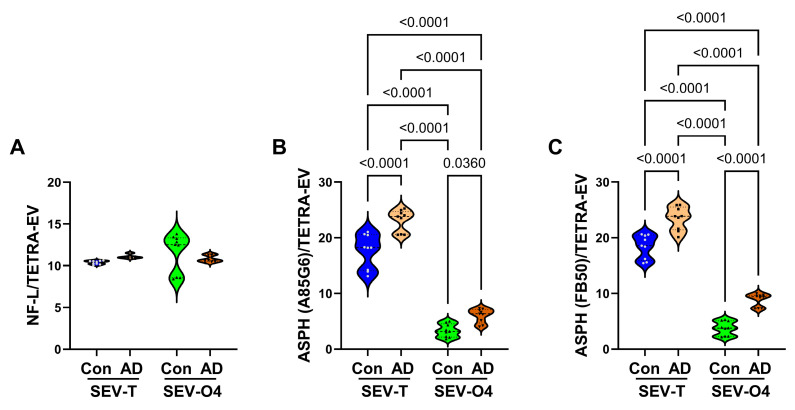
AD effects on neurofilament light chain (NfL) and aspartyl-asparaginyl-β-hydroxylase (ASPH) proteins. ASPH was detected with two different monoclonal antibodies: A85G6 and FB50. ELISAs measured immunoreactivity to (**A**) NFL, (**B**) ASPH (A85G6), and (**C**) ASPH (FB50) in unfractionated/total) (SEV-T) and O4+ (SEV-O4) EVs from control (n = 9) and AD (n = 9) cases. The results displayed reflect immunoreactivity normalized to tetraspanins measured with an antibody cocktail (CD9 + CD63 + CD81). Inter-group comparisons were made by ANOVA (see Table 3), and significant software-calculated *p*-values (*p* < 0.05) corresponding to post hoc Tukey tests are displayed.

*NfL and ASPH*: Neurofilament light chain (NfL) was measured because previous studies showed that the accumulation of NfL is a feature of axonal damage in various neurological diseases, including neurodegeneration [14,15,32]. Pathologically elevated levels of NfL have been detected in AD serum and cerebrospinal fluid [15,33], including in presymptomatic stages of disease [20]. Moreover, elevated levels of NfL were detected in neuronally derived EVs of patients with Parkinson’s disease [34] and in oligodendrocyte-selected EVs [35]. NfL immunoreactivity was investigated to determine if AD-related abnormalities were detectable in SEV-T or SEV-O4. ANOVA test revealed no significant inter-group differences. Correspondingly, the graphed results demonstrated similar levels of NfL immunoreactivity in control and AD SEV-T and SEV-O4 samples (Figure 9A).

ASPH is an insulin/insulin-like growth factor-responsive molecule expressed in the brain and is an important regulator of Notch pathway signaling. In addition to growth factor regulation, oxidative stress also drives ASPH via activation of hypoxia-inducible factor 1 (HIF-1α) [11]. ASPH immunoreactivity was investigated because AD is associated with increased oxidative stress in the CNS, and recent studies showed that ASPH immunoreactivity is detectable in SEVs. ANOVA tests demonstrated significant inter-group differences in ASPH immunoreactivity detected with the A85G6 and FB50 monoclonal antibodies (Table 3). The graphs showed significantly higher levels of ASPH (A85G6 and FB50) in AD relative to corresponding SEV-T and SEV-O4 samples (Figure 9B,C). In addition, the mean levels of ASPH in SEV-T samples were approximately fourfold higher than in the SEV-O4 samples (all *p* < 0.0001).

**Figure 10:**
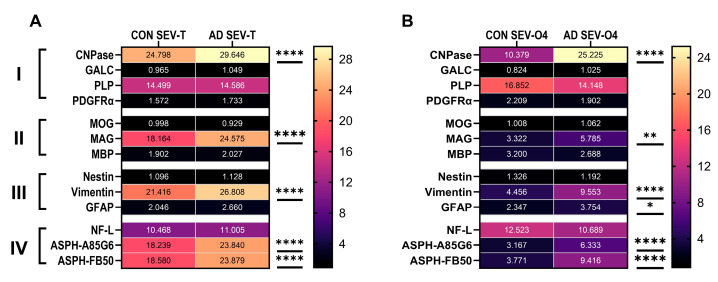
Heatmap summaries of AD effects on glial molecule immunoreactivity in (**A**) unfractionated (SEV-T) versus (**B**) O4+ (SEV-O4) EVs isolated from human serum. I—immature/non-myelinating oligodendrocyte glycoproteins; II—mature oligodendrocyte glycoproteins; III—astrocyte proteins; IV—neurofilament light chain and ASPH. The heatmaps were generated with GraphPad Prism 10.5. The color scales correspond to mean levels of immunoreactivity (displayed within the tiles). The data were analyzed by ANOVA and post hoc Tukey tests (**** *p* < 0.0001; ** *p* < 0.01; * *p* < 0.05).

*Comparative Heatmap Displays*: In addition to identifying potential non-invasive diagnostic aids for white matter degeneration in AD, the study aimed to determine whether CNS EV subset analysis would increase assay sensitivity. Heatmaps were used to compare the overall AD effects on glial protein expression in SEV-T and SEV-O4 samples (Table 4 and Figure 10).

The two-way ANOVA tests detected nearly identical AD effects in the SEV-T and SEV-O4 samples (Figure 10). Only one immature (CNPase) and one mature (MAG) oligodendrocyte/myelin glycoprotein were significantly elevated in both the unfractionated and O4+ SEVs. Among the astrocytic markers, vimentin was significantly elevated in both SEV sources, whereas increased GFAP immunoreactivity was selectively detected in SEV-O4, suggesting that O4 fractionated results may increase the sensitivity of assays designed to detect AD white matter degeneration. Finally, ASPH immunoreactivity was strikingly elevated in AD SEV-T and SEV-O4.

## 4. Discussion

The complexities of AD neurodegeneration extend well beyond accumulations of Aβ and pTau pathologies, and include white matter degeneration, microvascular disease, neuroinflammation, and impairments in energy metabolism [1]. Improved recognition of the breadth of AD pathology has diversified research on disease mechanisms, diagnostics, and therapeutics. For example, neuroinflammation has been addressed through the use of anti-inflammatory and antioxidant agents [36]. Dysregulated brain metabolism is being addressed by strategies that enhance glucose utilization and insulin signaling networks, including pharmaceutical [37,38,39,40,41] and physical exercise measures [42,43]. However, approaches for detecting and managing AD-related white matter degeneration still lag, in part because the mechanisms remain poorly understood.

Earlier studies showed that AD white matter degeneration was associated with progressive myelin loss, fiber rarefaction, and vasculopathy, beginning early in the course of the disease [1]. The most likely candidate cell types targeted in white matter degeneration include oligodendrocytes, astrocytes, and vascular endothelial cells [1]. Functional impairment of oligodendrocytes leads to myelin loss, which threatens the integrity of axons. At the same time, neuronal degeneration and retraction of cell processes lead to secondary myelin loss and synaptic disconnection. Vasculopathy with endothelial cell dysfunction compromises perfusion and delivery of nutrients needed for metabolic function, and it also leads to ischemic damage. Finally, these pathologies lead to activation of astrocytes and microglia, which drive neuroinflammatory responses, as well as promote scarring that could prevent efficient repair/regeneration. Herein, we examine how the expression of oligodendrocyte/myelin-associated glycoprotein mRNA transcripts is modified in AD brain tissue, and whether abnormalities in oligodendrocyte function can be detected in EVs isolated from serum. The efforts were further extended to determine whether sub-fractionation of oligodendrocyte-related serum EV populations would be needed to detect AD-related oligodendrocyte/myelin pathology.

Interest in EV liquid biopsy diagnostic approaches has grown exponentially over the past several years. Initially, the dominant focus was on malignancies, but more recently, data have emerged showing the utility of EV assays for detecting and monitoring chronic disease states, including neurodegeneration [10,22]. For example, recent studies have shown that typical AD biomarkers, including pTau and Aβ, are expressed in EVs [44,45]. Although EV assays are not specifically needed to measure impairments in serum Aβ secretion and pTau pathology, the approach provides opportunities to assess clustered pathologies and novel biomarkers linked to EV secretion in relation to neurodegeneration.

To characterize AD-associated white matter molecular pathology, we used quantitative RT-PCR analysis to measure mRNA expression corresponding to oligodendrocyte/myelin glycoproteins, with results normalized to housekeeping genes. Those analyses revealed significant AD-associated reductions in *PLP*, *MOG*, *MAG*, and *MBP*, and increased *VIM,* as well as a statistical trendwise upregulation of *GFAP.* These findings suggest that in AD, oligodendrocyte function is impaired at the mRNA level, whereas astrocyte functions are increased. Reduced oligodendrocyte/myelin glycoprotein mRNA expression in AD could result from oligodendrocyte loss due to injury, degeneration, or impaired function. Importantly, this molecular abnormality could account for the AD-associated reductions in myelin integrity [46,47]. The increased expression of astrocytic structural proteins likely reflects gliosis, a well-recognized feature of AD [1]. Increased gliosis contributes to neurodegeneration by driving neuroinflammation and can exacerbate ongoing damage [1].

A major limitation of AD research is the lack of feasible means to characterize and monitor white matter pathology using minimally invasive approaches. However, the implementation of serum-based EV assays provides opportunities for improvement. Recent studies have shown that in experimental models of Alzheimer’s disease or alcohol-related brain damage, white matter abnormalities in oligodendrocyte/myelin glycoprotein expression are reflected in SEVs [9,10].

In the present study, we characterized AD-associated alterations in oligodendrocyte/myelin glycoprotein and astrocytic structural proteins in unfractionated and O4+ selected EVs isolated from serum. The O4+ EV studies were used to determine if refinements were needed to enhance the sensitivity/specificity of EV assays for detecting white matter neurodegeneration. The studies demonstrated significant AD-associated increases in CNPase, MAG1, vimentin, and a trendwise increase in GFAP. However, the seemingly discordant reductions in several oligodendrocyte/myelin mRNAs vis-à-vis increased CNPase and MAG immunoreactivity could be explained by cell loss, coupled with pathophysiological states that compromise membrane integrity, leading to increased membrane breakdown and the attendant shedding of membrane glycoproteins into EVs. The absence of broad AD-associated alterations in EV oligodendrocyte/myelin glycoprotein immunoreactivity, despite significant reductions in their mRNAs, may have been due to limitations in immunoassay sensitivity, since significant AD effects were primarily observed in the more abundantly expressed molecules. Another consideration is that since the severity of AD was greater in the postmortem compared with the clinical cases, the extent of white matter pathology and oligodendrocyte loss/damage was also less severe. These matters could be clarified in future studies by performing paired analyses of brain and serum EV samples from the same cases. On the other hand, the finding of concordant AD-associated elevations in astrocytic markers in brain tissue and EVs supports the role of gliosis in neurodegeneration and highlights the potential use of this biomarker to detect and monitor neurodegeneration in AD.

Although NfL has been used as a non-invasive, serum-based marker of neurodegeneration in various diseases, including experimental models and human cases of AD [14,15,16,17,20], neurodegeneration-linked elevations in EVs have not been consistently detected, and may require cofactor damage such as traumatic brain injury [22]. Alternatively, neuronal subtype EV enrichment may be required to achieve sufficient sensitivity in SEV assays to measure the effects of neurodegeneration on NfL. Likewise, we failed to detect significant AD-associated increases in NfL in SEV-T or SEV-O4+.

ASPH immunoreactivity was detected with the A85G6 and FB50 monoclonal antibodies, which recognize the full-length and N-terminal truncated forms of the protein [48]. ASPH is expressed in white matter glial cells, particularly oligodendrocytes [12,13]. Its role in oligodendrocytes is likely linked to Notch pathway signaling [49], which regulates myelin homeostatic functions [50,51]. The results demonstrated striking elevations of ASPH immunoreactivity in both SEV-T and SEV-O4, confirming expression/localization in EVs and in oligodendrocyte-derived EVs. Given the known increased levels of oxidative stress in AD [52] and stimulatory effects of HIF-1α on ASPH [11], the AD-associated increases in SEV-ASPH immunoreactivity could serve as a biomarker of white matter injury/degeneration. While it is doubtful that this response is specific to AD, it could nonetheless aid in assessing the presence of oligodendrocyte/myelin/white matter pathology and, perhaps, in assessing their responses to treatments that effectively diminish white matter degeneration and bolster conductivity needed to enhance cognition.

## 5. Conclusions and Limitations

The main findings were that human brains (frontal lobes) with advanced stages of AD exhibited significant abnormalities in the expression of multiple glial mRNA transcripts marked by reduced PLP1, MOG, MAG, and MBP, and increased vimentin and GFAP. The clinical AD serum samples were obtained from individuals with earlier stages of AD neurodegeneration. EVs isolated from serum revealed significant AD effects on CNPase, MOG, MAG, Vimentin, GFAP, and ASPH. Other findings of note were as follows: (1) pooled tetraspanin immunoreactivity was found to be an excellent marker for normalizing EV immunoreactivity, because unlike HSP70 and RPLPO, its levels were not significantly modulated by AD status; (2) the immunoassay results obtained using SEV-T and SEV-O4+ were similar, suggesting that the analysis of subfractionated EVs may not improve sensitivity or sensitivity of assays used to detect white matter pathology in AD; (3) ASPH, a potent activator of both Notch signaling and HIF-1a, which are integrally linked to oligodendrocyte function and myelin integrity, was significantly increased in AD EVs; and (4) NfL immunoreactivity, a marker of neurodegeneration, was not significantly elevated in AD EVs, contrasting with earlier reports using serum-based assays in humans or experimental models [14,15,16,17,20].

Despite its interesting and provocative results, the study’s main limitations should be acknowledged to improve future research strategies. One noteworthy point is that the clinical diagnosis of AD was rendered using standard NINDS criteria, along with MMSE tests and clinical laboratory tests of Aβ_1–42_ and pTau. Unfortunately, at the time when the samples were obtained, pTau-(T307) was measured using a commercial ELISA. Current standards include assays for p-tau18, p-tau217, and p-tau231, which carry diagnostic weight [53], whereas p-tau307 is not currently used in serum-based AD assays. On the other hand, previous analysis of the same clinical cases demonstrated significantly elevated cerebrospinal fluid (CSF) levels of p-tau307 and CSF/serum p-tau307 ratios, supporting the diagnosis of early AD [31]. Unfortunately, no CSF samples were used in the present research.

Another limitation of the study was that the postmortem brain tissue and clinical serum EV assays were performed on samples from different disease stages. Future studies should use paired time-point brain tissue/serum samples to better correlate brain pathological changes with corresponding EV biomarkers. A final concern is that in the clinical cases, the controls were significantly younger than the AD group. Multivariate analysis of variance (MANOVA) was used to assess the effects of age on biomarker expression in both brain tissue and SEVs. MANOVA failed to detect significant effects of age on any of the white matter/glial biomarkers tested (Table S4). However, for the clinical samples, significant effects of age were observed for GALC, MOG, MBP, vimentin, GFAP, and NfL (Table S4), although AD-specific effects were not detected for GALC, MOG, or NfL (See Figure 6, Figure 7, Figure 8 and Figure 9). Moreover, although the age and AD-related directional shifts in vimentin and GFAP were similar between the mRNA (brain) and protein (SEV) studies, the results were discordant for oligodendroglial molecules, and age effects were observed for immunoreactivity but not for mRNA corresponding to the same molecules. Therefore, aging may have contributed to increased astrocyte activation in AD, whereas the alterations in oligodendrocyte protein/mRNA were more likely AD-driven rather than aging-dependent.

In conclusion, this study demonstrates the potential utility of minimally invasive EV assays to detect and monitor molecular abnormalities related to white matter degeneration in AD. Although the sample sizes were modest, the results suggest that subfractionation of EVs based on oligodendrocyte surface markers may not be required to detect white matter pathology, perhaps due to the narrow cellular distribution of oligodendrocyte/myelin glycoprotein expression in the body. Additional studies are needed to further assess the utility of broad versus fractionated EV-based assays for detecting neuronal, vascular endothelial, and metabolic markers of AD neurodegeneration.

## Figures and Tables

**Table 1:** Human postmortem brain cases.

Group	Braak Stage AD	# Cases	Age (Mean ± S.D.; Yrs)	Sex
Control	0–2	8	78.33 ± 11.16	4 Males; 4 Females
AD	5–6	8	84.01 ± 2.45	4 Males; 4 Females

**Table 2:** Human Serum EV study.

Group	# Cases	Age (Mean ± S.D.)	Sex	MMSE	Aβ_1–42_ (Serum)	pTau (pT307) (Serum)
Control	9	45.6 ± 11.18	5 M; 4 F	N.T.	54,415 ± 18,330	4271 ± 739
AD	9	67.5 ± 11.3	4 M; 5 F	21.9 ± 5.5	28,287 ± 3554	3581 ± 499
*t*-Test		*p* < 0.0001			*p* < 0.0001	*p* = 0.04

**Table 3:** ANOVA test results for ELISAs.

Protein	F-Ratio	*p*-Value
Tetraspanins	1.364	N.S.
HSP70	152.9	<0.0001
RPLPO	127.2	<0.0001
CNPase	122.4	<0.0001
GALC	1.765	N.S.
PLP	0.565	N.S.
PDGFRA	8.456	0.0003
MAG	143.7	<0.0001
MOG	3.17	0.037
MBP	16.68	<0.0001
Nestin	0.686	N.S.

ELISA results were analyzed by one-way ANOVA to compare inter-group differences in immunoreactivity in AD (n = 9) and control (n = 9) SEVs. N.S. = not significant.

**Table 4:** Two-way ANOVA EV ELISA results.

EV Source	AD-Factor F-Ratio	*p*-Value	Biomarker F-Ratio	*p*-Value	AD × Biomarker F-Ratio	*p*-Value
SEV-T	118.6	<0.0001	719.4	<0.0001	12.77	<0.0001
SEV-O4+	180.5	<0.0001	340.7	<0.0001	51.25	<0.0001

Table 4: Duplex ELISA results corresponding to immunoreactivity, measured in total, and O4+ selected EVs isolated from serum were analyzed by two-way ANOVA to analyze the effects of AD, biomarkers, and AD x biomarker interactions. The calculated F-ratios and *p*-values are tabulated. Significant effects (*p* ≤ 0.05) are highlighted with bold font. The results of post hoc Šídák’s multiple-comparison tests are shown in Figure 10. DFn, DFd = 12,208 for SEV-T and SEV-O4+.

## Data Availability

The original contributions presented in this study are included in the article/Supplementary Material. Further inquiries can be directed to the corresponding author.

## Supplementary Materials

The following supporting information can be downloaded at: https://www.mdpi.com/article/10.3390/biomedicines14010251/s1, Table S1: Glial Genes Included in Custom PCR Array; Table S2: PCR Primer Pairs Used in the Targeted Human Glial Gene Expression Array; Table S3: Commercial Antibodies, Sources, and Validation References; Table S4: Multivariate analysis of variance (MANOVA) Report.

## Abbreviations

The following abbreviations are used in this manuscript:

AD	Alzheimer’s Disease
APOE	Apolipoprotein E
ASPH	Aspartyl-Asparaginyl-β-Hydroxylase
CD63	Tetraspanin-30
CD81	Tetraspanin-28
CD9	Tetraspanin-29
CNP	2′,3′-Cyclic Nucleotide 3′ Phosphodiesterase
CNS	Central Nervous System
ELISA	Enzyme-Linked Immunosorbent Assay
EV	Extracellular Vesicles
GALC	Group-Specific Component Vitamin D Binding Protein
GFAP	Glial Fibrillary Acidic Protein
HIF-1α	Hypoxia-Inducible Factor 1
HPRT	Hypoxanthine Phosphoribosyltransferase
HSP70	Heat Shock Protein 70
MAG	Myelin-Associated Glycoprotein
MBP	Myelin Basic Protein
MOG	Myelin Oligodendrocyte Glycoprotein
NfL	Neurofilament Light Chain
PDGFR-α	Platelet-Derived Growth Factor-Alpha
PLP	Proteolipid Protein
RPLPO	Large Acidic Ribosomal Protein
SAPE	Streptavidin-Conjugated R-Phycoerythrin
SEV-O4	Serum-Derived Extracellular Vesicles, O4+ Fractionated
SEV-T	Serum-Derived Extracellular Vesicles, Unfractionated
