# Supplementary material for: Alzheimer’s Disease-Associated Molecular Abnormalities in White Matter Glia and Related Pathologies Detected in Unfractionated and O4-Selected Serum Exosomes Using a Liquid Biopsy Approach
Source: Biomedicines. 2026 Jan 22;14(1):251. doi: 10.3390/biomedicines14010251 (PMC12838737; doi:10.3390/biomedicines14010251)
Supplement: Supplementary file 1 [file biomedicines-14-00251-s001.zip › biomedicines-4081713-supplementary.pdf]

Supplementary Table S1: Glial Genes Included in Custom PCR Array

Abbreviation	Full Name	Gene Names	Product Functions
CNP	2',3'-cyclic nucleotide 3' phosphodiesterase	CNPase, EC, CNP1	Myelin-associated marker of oligodendrocytes and Schwann cells. It may play an important role in the development of myelin membranes and sustain axonal integrity.
GALC	Group-specific component Vitamin D Binding protein; Gc-globulin	GBD, DBP/GC, VDB	Multifunctional member of the albumin family, found in plasma, ascites fluid, cerebrospinal fluid, and on cell surfaces. Binds Vitamin D and plasma metabolites and transports them to target tissues.
GFAP	Glial Fibrillary Acidic Protein	Intermediate Filament Protein	Astrocyte intermediate filament cytoskeletal protein.
MAG	Myelin Associated Glycoprotein	GMA	Glycoprotein facilitating sialic acid-dependent cell-cell interactions between neuronal and myelinating cells. Found on oligodendrocytes and Schwann cells.
MBP	Myelin Basic Protein	Myelin A1 Protein	A major component of myelin sheaths in both oligodendrocytes and Schwann cells. Aids in the formation and stabilization of myelin membranes.
MOG	Myelin Oligodendrocyte Glycoprotein	MOGIG2	Expressed on the oligodendrocyte cell surface and the outer surface of myelin sheaths. Possibly involved in the completion or maintenance of myelin sheaths.
NES	Nestin		An intermediate protein that promotes the disassembly of phosphorylated vimentin during mitosis. Required for survival, renewal, and mitogen-stimulated proliferation of neural progenitor cells.
PDGFRA	Platelet Derived Growth Factor Receptor, alpha polypeptide	CD140A, Alpha Platelet-Derived Growth Factor Receptor	Cell surface tyrosine protein kinase receptor required for skeletal development and cephalic closure during embryonic development. Survival factor for oligodendrocyte progenitor cells.
PLP1	Proteolipid Protein 1	PLP, SPG2	Transmembrane proteolipid protein, dominant in CNS myelin. May be involved in compaction, stabilization, maintenance of myelin sheaths, oligodendrocyte development, and axonal survival.
VIM	Vimentin	CTRCT30	Class-III intermediate filament that maintains cell shape and cytoplasm integrity and stabilizes cytoskeletal interactions. May be involved in peripheral nerve myelination.

Supplementary Table S2: PCR Primer Pairs Used in the Targeted Human Glial Gene Expression Array

Abbreviation	Gene Name	Forward/Reverse	Sequence (5′→3′)	Position (mRNA)	Amplicon (bp)
CNP	2',3'-Cyclic Nucleotide 3' Phosphodiesterase	FOR	TATGTAAAGCGGCCGGGCT	25	166
CNP		REV	TCTTGGGCAGGAATGTGTGG	190	
GALC	Group-Specific Component (Vitamin D Binding Protein)	FOR	TGCTGCTTATGGGGAGAAGAA	884	142
GALC		REV	AGAGGCAGACTCACAGCATT	1025	
GFAP	Glial Fibrillary Acidic Protein	FOR	GAGGGACAATCTGGCACAGG	486	178
GFAP		REV	GAACCGGATCTCCTCCTCCA	663	
MAG	Myelin Associated Glycoprotein	FOR	GTGTCACCCGAGGATGATGG	1342	167
MAG		REV	TTGGACTTCACCACGCACAG	1508	
MBP	Myelin Basic Protein	FOR	GCTGCAGAGACAGAGAGGAC	1081	156
MBP		REV	GCTCCCACATGTAGTAAGCCA	1236	
MOG	Myelin Oligodendrocyte Glycoprotein	FOR	GGGCAACATGCCTGCTTTAG	1234	172
MOG		REV	AGCACCTAGCTTGTTTGTTTGT	1405	
NES	Nestin	FOR	AGCGTTGGAACAGAGGTTGG	853	119
NES		REV	GAGCGATCTGGCTCTGTAGG	971	

PDGFRA	Platelet-Derived Growth Factor Receptor, Alpha Polypeptide	FOR	TGTGGGACATTCATTGCGGA	282	125
PDGFRA		REV	AAGCTGGCAGAGGATTAGGC	406	
PLP1	Proteolipid Protein 1	FOR	AGAACAGACTGGCCTGAGGA	1946	174
PLP1		REV	CCCCCATGAAATGAGCACCA	2119	
VIM	Vimentin	FOR	AGGAAATGGCTCGTCACCTTC	1534	191
VIM		REV	GTGGGTATCAACCAGAGGGAG	1724	

Primer pairs used in a targeted qRT-PCR array.

Supplementary Table S3: Commercial Antibodies, Sources, and Validation References

Antibody Targets	Source	Monoclonal/ Polyclonal	Stock (mg/ml)	µg/ml or Dilution	Commercial Source	RRID# *
CD9 (Tetraspanin-29)	Rabbit	Polyclonal	8.66	0.5	ABclonal, Woburn, MA	A1703
CD63 (Tetraspanin-30)	Rabbit	Polyclonal	1.03	0.5	ABclonal, Woburn, MA	A5271
CD81 (Tetraspanin-28)	Rabbit	Polyclonal	1.76	0.5	ABclonal, Woburn, MA	A5270
HSP70 (Heat Shock Protein 70)	Rabbit	Polyclonal	1.84	0.5	ABclonal, Woburn, MA	A0284
RPLPO (Large acidic ribosomal protein)	Mouse	Monoclonal	0.1	0.1	Santa Cruz, Dallas TX	ab10738968
CNPase (11-5B) (2',3'-cyclic nucleotide 3' phosphodiesterase)	Mouse	Monoclonal	1.0	2.0	Abcam, Boston, MA	ab6319
GALC (Group-specific component Vitamin D Binding protein; Gc-globulin)	Rabbit	Polyclonal	1.0	2.0	Abcam, Boston, MA	ab83752
PLP (Proteolipid Protein 1)	Rabbit	Polyclonal	Serum	1:2000	Abcam, Boston, MA	ab28486
PDGFRA (Platelet-derived growth factor receptor, alpha polypeptide)	Rabbit	Polyclonal	1.0	1.0	Abcam, Boston, MA	ab61219
MAG1 (Myelin-Associated Glycoprotein 1)	Mouse	Monoclonal	0.5	0.25	Abcam, Boston, MA	ab89780

MOG (Myelin Oligodendrocyte Glycoprotein)	Rabbit	Polyclonal	1.0	2.0	Abcam, Boston, MA	ab32760
MBP (Myelin basic protein)	Rabbit	Polyclonal	1.0	2.0	MilliporeSigma, Burlington, MA	M3821
Nestin	Rabbit	Polyclonal	Serum	1:2000	Abcam, Boston, MA	ab27952
Vimentin	Mouse	Monoclonal	1.0	2.5	Abcam, Boston, MA	ab8978
GFAP (Glial Fibrillary Acidic Protein)	Goat	Polyclonal	0.5	0.5	Abcam, Boston, MA	ab53554
NfL (Neurofilament light chain)	Rabbit	Polyclonal	0.6	0.6	Abcam, Boston, MA	12998-1-AP
A85G6 (Aspartyl-Asparaginyl-β-Hydroxylase; ASPH)-	Mouse	Monoclonal	1.3	1.3	Invitrogen (Thermo Fisher Scientific	Reference **
FB50 (Aspartyl-Asparaginyl-β-Hydroxylase; ASPH)	Mouse	Monoclonal	0.845	0.845	Invitrogen (Thermo Fisher Scientific	Reference **

*RRID= Research Resource Identifier; References refer to literature citations with validations of RPLPO antibody.

** Antibody validation was by Rolf Carlson, Liver Research Center, Brown University Health, Providence, RI

Supplementary Table S4: Multivariate analysis of variance (MANOVA) Report

Factor	Test value	F-Ratio	p-value	Decision
		Frontal Lobe White Matter (mRNA Assay)		
Pillai's Trace	5.958	0.19	1.00	Accept
ACTIN	1.369589	5.13	0.101911	Accept
HPRT1	0.030115	1.56	0.398544	Accept
CNP	0.001976	1.87	0.335380	Accept
GALC	0.002810	0.94	0.606865	Accept
PLP1	136.086866	0.51	0.837431	Accept
PDGFRA	0.001115	2.94	0.203151	Accept
MOG	0.103817	0.58	0.796661	Accept
MAG	0.327949	1.33	0.461260	Accept
MBP	4121.342040	3.07	0.193092	Accept
NES	0.000024	0.55	0.813994	Accept
VIM	0.121178	0.37	0.918051	Accept
GFAP	54.139631	3.24	0.181161	Accept
		SEV-Total (ELISA Tests)		
Pillai's Trace	5.958	0.19	1.00	Accept
Tetraspanin	0.002799	0.78	0.693778	Accept
HSP70	0.002694	0.86	0.640300	Accept
RPLPO	447611395	1.01	0.535724	Accept
CNPase	0.036541	2.38	0.121081	Accept
GALC	0.000776	14.01	**0.000750**	**Reject**
PLP1	0.027114	1.10	0.480730	Accept
PDGFRA	0.000593	3.14	0.062546	Accept
MOG	0.000311	4.15	**0.030051**	**Reject**
MAG	0.014816	0.50	0.896732	Accept
MBP	0.001358	11.08	**0.001597**	**Reject**
Nestin	0.030985	1.01	0.534193	Accept
Vimentin	0.013750	4.83	**0.019703**	**Reject**
GFAP	0.023411	5.05	**0.017361**	**Reject**
NfL	0.232079	4.16	**0.029966**	**Reject**
ASPH-A85G6	14.229918	0.56	0.859012	Accept
ASPH-FB50	10.616557	0.94	0.583129	Accept

The Number Cruncher Statistical Software (NCSS; v2021) was used for one-way MANOVA to examine the effect of age on the expression of glial or housekeeping mRNA transcripts in the brain and proteins in serum extracellular vesicles (SEV). The MANOVA Pillai's Trace test result for age effects on glial and housekeeping gene expression in brain tissue was not significant (F195,39)=0.10 (N.S.). The MANOVA Pillai's Trace test result for age effects on glial and housekeeping protein immunoreactivity in SEVs was not statistically significant (F(352,112)=0.19; N.S. The Wilks Lambda and Hotelling-Lawley trace tests were also not statistically significant. Follow-up univariate tests revealed no significant differences for any of the mRNAs in brain tissue (DF1=15;DF2=3), but significant effects of age on GALC, MOG, MBP, Vimentin, GFAP, and NfL immunoreactivity in SEVs (DF1=22; DF2=7). However, none

of the significant age effects on glial proteins were associated with age effects on the corresponding mRNA transcripts, several significant age-related effects showed no significant inter-group differences by two-way ANOVA (NfL, GALC), and several non-significant age-effects showed significant differences between the AD and control groups by two-way ANOVA (HSP70, RPLPO, CNPase, PLP1, MAG, ASPH-A85G6, ASPH-FB50).
